# Trying times

**DOI:** 10.4103/0971-3026.50815

**Published:** 2009-05

**Authors:** Bhavin Jankharia

**Affiliations:** Editor-in-Chief, The Indian Journal of Radiology and Imaging, Bhaveshwar Vihar, Sardar V P Road, Mumbai, India. E-mail: editor@ijri.com

Most people are now tired of hearing the phrases, “global recession,” “meltdown,” etc. India does not seem to be as affected as some of the other Western countries, but that's no consolation to us.

We know there is a recession, especially when radiology companies start pulling out their advertisements. Three of them have already done so and the others are playing hardball. New companies are just not advertising. When a journal such as ours works on breaking even with revenues from advertising taking care of the expenses, a reduction in advertising revenue can be quite a problem. Though we have tried to sort this out in the best way possible and are aggressively going after new advertisers, I appeal to all the readers that if you see any company not represented in this issue and if you know anyone in that company in a decision-making capacity who you can influence in making a decision to advertise in the IJRI, please try your best to make sure that the company advertises in the journal. Please do not hesitate to contact any of us in the Editorial team.

## The benefits?

1. A high readership of more than 6500 people reading the advertisement
2. Longer shelf life in libraries, etc., where the advertisements continue to be seen long after the issue has been published, as against ads in other business magazines, where they have no shelf life.

This month's issue focuses on Breast Imaging with Dr. Bijal Jankharia as the Guest Editor. We will be carrying this theme for the next three issues till November 2009.

We are also starting a special series of invited articles on Tuberculosis from next month, beginning with an article on musculoskeletal tuberculosis. Articles on tuberculosis will be fast tracked for the whole of this year, especially, review articles that focus on specific issues or organ systems.

We have dropped the Medicolegal section due to lack of reader interest. Perhaps at a later date, when we can have a more focused approach to medicolegal problems in radiology, we may reintroduce this subject.

Apart from the special focus on Breast, this month's issue specifically focuses on Head and Neck, Neuro and Musculoskeletal Radiology with a combination of pictorial essays, original articles, and case reports. Perhaps it is also a sign of the times that five articles in this issue are from authors who reside outside the country.

We don't have enough reader feedback either through the website, or via e-mail (editor@ijri.org), or through snail mail. Good reader feedback either related to the articles published or on general issues relevant to radiologists and the IJRI may be published as “Letters-to-the-Editor.” Without reader feedback, it is difficult to gauge how relevant the IJRI and its various sections are to the radiologists who read the journal.

Happy reading!

